# Reverse correlating trustworthy faces in young and older adults

**DOI:** 10.3389/fpsyg.2013.00592

**Published:** 2013-09-05

**Authors:** Catherine Éthier-Majcher, Sven Joubert, Frédéric Gosselin

**Affiliations:** ^1^Centre de recherche en Neuropsychologie Expérimentale et CognitionMontréal, QC, Canada; ^2^Centre de recherche de I'Institut universitaire de gériatrie de MontréalMontréal, QC, Canada; ^3^Département de psychologie, Université de MontréalMontréal, QC, Canada

**Keywords:** trustworthiness judgments, facial expressions, aging, social cognition, reverse correlation

## Abstract

Little is known about how older persons determine if someone deserves their trust or not based on their facial appearance, a process referred to as “facial trustworthiness.”In the past few years, Todorov and colleagues have argued that, in young adults, trustworthiness judgments are an extension of emotional judgments, and therefore, that trust judgments are made based on a continuum between anger and happiness (Todorov, 2008; Engell et al., 2010). Evidence from the literature on emotion processing suggest that older adults tend to be less efficient than younger adults in the recognition of negative facial expressions (Calder et al., 2003; Firestone et al., 2007; Ruffman et al., 2008; Chaby and Narme, 2009). Based on Todorov';s theory and the fact that older adults seem to be less efficient than younger adults in identifying emotional expressions, one could expect that older individuals would have different representations of trustworthy faces and that they would use different cues than younger adults in order to make such judgments. We verified this hypothesis using a variation of Mangini and Biederman's (2004) reverse correlation method in order to test and compare classification images resulting from trustworthiness (in the context of money investment), from happiness, and from anger judgments in two groups of participants: young adults and older healthy adults. Our results show that for elderly participants, both happy and angry representations are correlated with trustworthiness judgments. However, in young adults, trustworthiness judgments are mainly correlated with happiness representations. These results suggest that young and older adults differ in their way of judging trustworthiness.

## Introduction

Recognizing someone';s intentions based on available facial information is a task performed on a daily basis. When meeting a new person, one of the most important social judgments is trust, which can subsequently determine the course of social interactions and prevent potentially risky encounters. Surprisingly, little is known about the evolution of facial trustworthiness processing during the course of aging. To our knowledge, only one study has directly addressed that question and has found age differences in bold activity related to trust judgments (Castle et al., 2012). It seems particularly worthwhile to pay attention to trustworthiness judgments in elderly populations as studies have shown that older adults are more vulnerable to fraud than their younger counterparts (Templeton and Kirkman, 2007; Alves and Wilson, 2008) and changes in trustworthiness judgments may be one of the contributing factors to this increased susceptibility. For instance, Tehan and Blanchard-Fields (2008) tested a group of young adults and a group of elderly on their ability to detect deceit using interviews (crime and opinion topics) presented in three conditions: audio only, visual only and audio-visual. Their results show that older adults are, overall, less efficient than younger adults in detecting deceit. Moreover, in the visual and audio-visual crime interviews, they show less benefit of the visual information than younger adults. The authors suggest that their results could be explained by a difference between younger adults and older adults in their ability to recognize facial expressions of emotions, particularly negative facial expressions of emotions such as fear and shame. This hypothesis finds support in the emotion recognition literature regarding normal aging. Indeed, many studies (although not all, see Mienaltowski et al., 2013) have shown that older individuals are less efficient in recognizing emotional expressions (Calder et al., 2003; Sullivan and Ruffman, 2004a,b; Firestone et al., 2007; Sullivan et al., 2007; Chaby and Narme, 2009). This reduced efficiency seems to affect mainly visual representations of emotions, more specifically the ability to recognize facial expressions of emotions (Phillips et al., 2002; Keightley et al., 2006). Recently, a meta-analysis covering 17 studies on facial expression recognition in aging has shown that anger, sadness and fear are less accurately recognized in elderly adults than in younger adults, although disgust seems to be equally or even better recognized in elderly participants when compared to younger adults (Ruffman et al., 2008).

Despite the poor amount of literature concerning trustworthiness judgments in elderly, quite a few studies have been published in the past few years in regards to this type of judgment in young adults, showing a growing interest in understanding the underlying processes of social judgments in this population. Even though faces are complex stimuli, it seems that social judgments based on faces can be achieved very easily. For instance, Willis and Todorov (2006) have shown that judgments of trust based on faces can be made quickly. In fact, exposing a face for 100 ms is sufficient for one to formulate a first impression on that face, and this first impression seems to be long-lasting. Nevertheless, a question persists regarding how we process such complex judgments in such a short time. Todorov et al. (2008) have suggested that the use of specific regions of the face contributes to making the decision of trusting someone or not. They reported that the judgment of trustworthiness mainly relies on four different facial features: the inner eyebrows, cheekbones, chins and nose sellion. A reverse correlation study by Dotsch and Todorov (2012) later established a relationship between trustworthy representations and untrustworthy representations in faces. Indeed, they showed that the representation of a trustworthy face corresponds to the opposite representation of an untrustworthy face, suggesting a continuum between trustworthiness and untrustworthiness representations. Moreover, the use of reverse correlation has allowed to identify the facial features used in determining whether a face is trustworthy or not, and to demonstrate that the eyes, mouth, eyebrows and hair regions are of particular importance in this type of decision.

To better understand trustworthiness judgments, Oosterhof and Todorov (2008) have developed a 2D model of face evaluation based on behavioral studies and computer modeling. This model suggests that in order to decide whether a face looks trustworthy or not, one would first judge that face on two dimensions: valence and dominance. In this model, valence evaluation is defined as an overgeneralization of perception of facial cues signaling whether to approach or avoid a person, whereas dominance evaluation is defined as an overgeneralization of perception of facial cues signaling the physical strength/weakness of the person. Oosterhof and Todorov (2008) have demonstrated that, in judging faces on 14 different traits, the trustworthiness judgment correlated highly with the valence of a face (0.94), and therefore was sufficient to summarize the evaluative information present in all other trait judgments. In other words, they argued that in situations where no other context is provided, judging a face on trustworthiness is a reflection of inferences about the positivity/negativity of a face.

Following these findings, Todorov';s group formulated the hypothesis that trustworthiness judgments might be an extension of emotional judgments in a context where we need to decide whether to approach or to avoid a person showing a neutral face, without any emotional cue. They first addressed this question using their model of face evaluation, where they took the faces judged as the most trustworthy/untrustworthy and exaggerated the features of the face found to influence the judgment of trustworthiness. They then asked participants to classify these “extreme” trustworthy/untrustworthy faces into categories corresponding to the six basic emotions. When relating the trustworthiness level of these faces with the category in which the faces were classified, the only responses above chance were the ones where faces were categorized as angry, neutral and happy. Based on this information, Todorov (2008) has argued that, in young adults, trustworthiness judgments are an extension of emotional judgments, and therefore that the judgment of trustworthiness is based on a continuum between anger and happiness. Todorov';s group further strengthened this theory by testing their hypothesis in behavioral studies using three different paradigms. In the first study, Oosterhof and Todorov (2009) used a dynamic stimulus paradigm in which participants saw a dynamic transformation between a neutral face and a happy or angry face and were asked to judge the intensity of the final face';s expression. Using the same stimuli, the authors manipulated the degree of trustworthiness of the faces presented. Their results suggested that changes on the trustworthiness continuum corresponded to slight changes resembling emotional expressions. In another study, Said et al. (2009) used a Bayesian network classifier, trained to detect emotional expressions, to show that neutral faces were classified based on trait inferences and that positive valence resembles happiness expressions, whereas negative valence resembles fear or disgust expressions. Finally, Engell et al. (2010) used a behavioral adaptation paradigm and found that adapting to angry or happy facial expressions causes trustworthiness evaluations of subsequently rated neutral faces to increase or decrease, respectively. These three studies thus support the existence of a relationship between trustworthiness judgments and emotional judgments, and, more specifically, a link between happiness, anger and trustworthiness judgments. Furthermore, this theory finds support in the emotion recognition literature, which provides evidence that the structures used to identify basic emotions might also be involved in the process of social judgments. For instance, studies of patients presenting lesions to the amygdala have revealed that these patients are not only impaired in the recognition of negative emotions, but also tend to judge faces as more trustworthy than controls (Adolphs et al., 1994, 1998).

In sum, evidence from behavioral studies on trustworthiness judgments in young adults and patient studies seem to relate social judgments to emotional judgments. Considering that older adults seem to be less efficient than younger adults in identifying emotional expressions, one could expect that older persons would have different mental representations of trustworthy faces. Older adults might also use different cues than younger adults in order to make trust judgments. We sought to explore this hypothesis in two groups of participants: young and older adults. Contrary to previous studies on facial trustworthiness, such as those of Todorov and colleagues, we asked participants to judge trustworthiness in a specific context (i.e., money investment) in order to eliminate context as a potential source of variability—especially across age groups—from our results. We employed a classification image method, capable of revealing properties of mental representations of trustworthiness, happiness, and anger. In the past 10 years or so, such methods have been used to better understand the internal representations underlying face recognition (e.g., Gosselin and Schyns, 2001, 2003; Schyns et al., 2002; Mangini and Biederman, 2004; Sekuler et al., 2004; Adolphs et al., 2005; Smith et al., 2005; Blais et al., 2012). More recently, as illustrated by this special issue, classification images have even been used to examine mental representations of the social brain: the internal representation of facial emotion recognition across cultures (Dotsch et al., 2008; Jack et al., 2011, 2012) and the identification of trustworthy and of untrustworthy faces (Dotsch and Todorov, 2012).

## Methods

In this study, trustworthiness judgments were investigated by applying a variation of Mangini and Biederman's (2004) reverse correlation technique. This method consists of asking participants to do a discrimination task on faces to which noise is added. In Mangini and Biederman';s experiments, noise patterns were created based on 6 different orientations and 2 different phases and participants were asked to categorize one face regarding gender, expression and identity. Our method was different in three ways. First, instead of presenting only one stimulus and asking participants to categorize this unique stimulus, we presented two stimuli and asked participants to choose between the two stimuli. This way, the participants'; choices rely only on the different influence of noise on the same faces (see also Dupuis-Roy and Gosselin, 2007; Dotsch and Todorov, 2012). Second, the patterns of noise consisted of Gaussian white noise. Third and, finally, the underlying faces were different on each trial and were not chosen to be neutral on the combination of trustworthiness, happiness and anger, which would have been difficult, if not impossible, to achieve. Instead, different faces of *women* expressing a neutral emotion were presented on each trial. This bank of faces was chosen because it contains faces of young and older adults. We limited ourselves to women to eliminate the variability in judgments (anger, and, possibly, trust) attributable to interactions with gender.

### Participants

Thirty-seven young adults (13 men) between 19 and 29 years old (median = 21 years, inter-quartile range = 4, mean = 22 years, standard deviation = 2.7) and 25 older adults (12 men) between 56 and 74 years old (median = 64 years, inter-quartile range = 8.5, mean = 65 years, standard deviation = 5.6) took part in this experiment. Young participants were recruited on campus whereas elderly participants were recruited via a bank of normal elderly participants from the Centre de recherche de l';Institut universitaire de gériatrie de Montréal (CRIUGM). For the group of older adults, all participants were assessed with a battery of neuropsychological tasks, in order to exclude the presence of major cognitive deficits. Performance on these tests is summarized in Table 1. A vast majority of participants from the two groups were Caucasian and the others had been living in Montreal since a very early age. All participants had normal or corrected to normal vision. This was verified using standard questions to ensure that the older participants did not suffer from common visual conditions (such as cataract, glaucoma, macular degeneration, etc.).

**Table 1:** **Neuropsychological results of elderly participants**.

**Test**	***N***	**Mean**	***SD***
MoCA	24	26.96	1.92
MMSE	19	29.12	0.78
Digit span			
*Forward*	24	6.5	5.4
*Backward*	24	1.3	1.0
Benton faces	25	45.96	4.08
Benton lines	25	24.77	3.27
VOSP	25	19.69	0.47
Trails A (time)	25	36.68	14.96
Trails B (time)	25	69.64	22.41
BNT—short form	25	14.04	1.86
Buschke—1st recall	22	9.6	2.41
Buschke—2nd recall	22	11.65	2.71
Buschke—3rd recall	22	12.55	2.48
Buschke—delayed recall	22	13.55	2.37
RCFT—copy	25	33.88	1.47
RCFT—immediate recall	25	15.13	5.54
RCFT—delayed recall	24	16.71	3.72

### Stimuli

On each trial in a block, one of 150 grayscale face pictures (256 × 256 pixels) was picked (without replacement). All these images depicted a front-view and eyes-open Caucasian woman face aged between 20 and 70 years old. The face depicted a neutral expression. The faces were aligned on 12 handpicked, easily-identifiable facial landmarks (four landmarks on each eye and four on the mouth) using linear conformal transformation. Two Gaussian noise fields of 128 × 128 pixels rescaled at 256 × 256 pixels (with the nearest-neighbor algorithm) were added to that face to produce two face stimuli. Thus, for each trial, two images (image width = 6.8° of visual angle; face width = about 3.8° of visual angle), only differing by the pattern of noise added to the original image, were presented side-by-side on the screen (3.6° of visual angle apart) for 3 s (see Figure 2 for an example of stimulus). All stimuli were presented on CRT monitors (1024 × 768 pixels), calibrated using a Samsung SyncMaster753df photometer to allow linear manipulation of luminance. The resulting corrected table contained 137 luminance levels, ranging from 0.31 to 107 cd/m^2^. On average, the RMS of the base images was equal to 0.2176 (*SD* = 0.0181) and that of the noise fields to 0.1999 (*SD* = 0.0011). The background luminance was equal to 53.65 cd/m^2^. The refresh rate was 60 Hz. Distance to the screen was maintained at 50 cm (using a chinrest) during the whole experiment.

### Procedures

**Figure 1:**
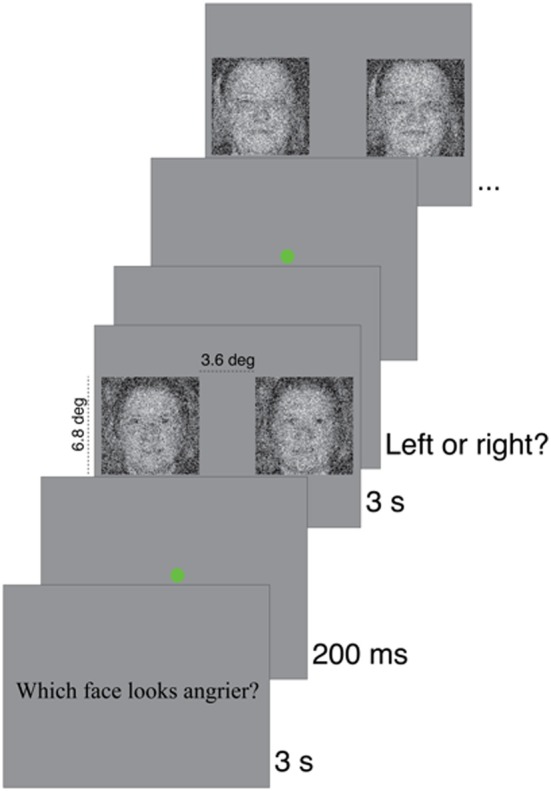
**Sequence of events in the experiment**. The question appeared on the first frame of a block. Then, a green dot served as a fixation point and appeared on the screen for 200 ms. The stimuli then appeared for three seconds after which a choice was required. This sequence of events was repetead for the 150 trials of the block.

The experimental programs were run on a PC computer in the Matlab (Mathworks™) environment and used functions from the Psychophysics toolbox (Brainard, 1997; Pelli, 1997). Participants completed two blocks of 150 trials for each of the three conditions (trustworthiness, happiness, and anger). The order of presentation of the conditions was counter-balanced across subjects. At the beginning of each block, the question to which the participant had to answer for that specific block appeared on the screen (e.g., “Which face looks angrier?”). For the trustworthiness condition, the notion of trust was put in a context of money investment (i.e., “If you had a big amount of money to invest, who would you trust the most with your money?”). We feared that the default trustworthiness contexts would be much more dissimilar between age groups than within them. As we were not interested in these default trustworthiness contexts *per se*—we were rather interested in comparing implicit representations of faces expressing trustworthiness in comparable contexts—we provided this money investment context, which is usual for both age groups. In contrast, Todorov and colleagues did not contextualize facial trustworthiness in their studies. Participants saw the stimuli for 3 s, after which they had to choose, by pressing right or left keys on the keyboard, which one of the two stimuli presented seemed the most trustworthy/happy/angry (see Figure 1). Participants were told that the task was difficult, to do their best to answer correctly, based on their first general impression, without taking too much time.

### Results

For each condition and for each individual, we subtracted the sum of the standardized Gaussian noise fields of 128 × 128 pixels of all rejected stimuli from the sum of the standardized Gaussian noise fields of 128 × 128 pixels of all chosen stimuli (e.g., Dupuis-Roy and Gosselin, 2007), thus obtaining a total of three individual classification images (CI) of 128 × 128 pixels. Then, we smoothed the CIs using a Gaussian filter (*SD* = 3 pixels), and transformed these smooth CIs into *Z*-score planes by dividing them by the square root of the number of standardized Gaussian noise fields that went into their construction. Not a single pixel of these individual CIs actually attained statistical significance (Pixel test, two-tailed, search region = 4275 pixels, *p* > 0.05; for details, see Chauvin et al., 2005).

**Figure 2:**
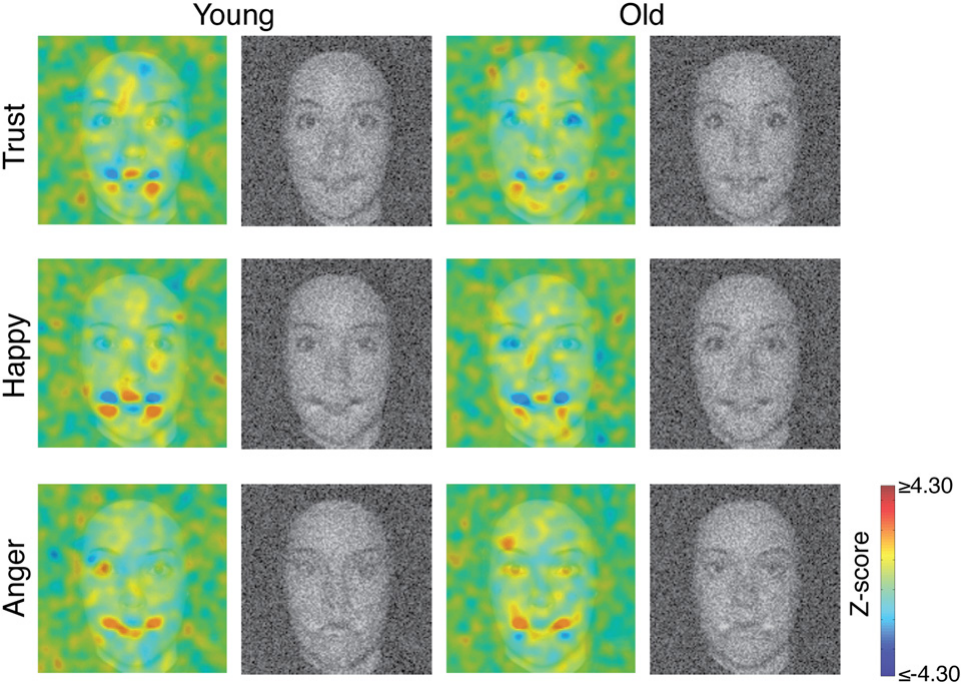
**Colored images represent smooth group-judgment classification images transformed into *z*-scores superimposed on a grayscale face**. Grayscale images represent raw group-judgment classification images added to a grayscale face with the same range of values.

To increase signal-to-noise ratio, we decided to combine all individual CIs per age group and per judgment. However, such group CIs are meaningful only if there is good agreement between the combined individual CIs. Therefore, to evaluate this agreement, we calculated the Pearson correlations between every individual CI';s and the corresponding group CI';s, restricting the computation to the union of areas that attained statistical significance in all group CI';s (all judgments and age groups confounded)—a total of 297 pixels. Mean correlations were 0.33 (quartiles: 0.24, 0.35, and 0.43; 30/31 correlations were positive), 0.22 (quartiles: 0.11, 0.26, and 0.37; 28/31 correlations were positive), and 0.18 (quartiles: 0.13, 0.20, and 0.26; 26/31 correlations were positive), respectively, for the happiness, anger, and trustworthiness CI';s in younger adults; and mean correlations were 0.63 (quartiles: 0.54, 0.65, and 0.78; all correlations were positive), 0.53 (quartiles: 0.36, 0.55, and 0.73; all correlations were positive), and 0.46 (quartiles: 0.33, 0.51, and 0.63; 24/25 correlations were positive), respectively, for the happiness, anger, and trustworthiness CI';s in older adults. This suggests indeed a high reliability across participants. We thus summed the individual CIs within each condition and subject group (see Figure 2), obtaining a total of six group CIs. We transformed these group CIs into *Z*-scores planes by dividing them by the square root of the number of individuals in the appropriate subject group. In Figure 2, the *Z*-scored group CIs are superimposed on a grayscale face to help interpretation. The bright red (*Z*-score ≥ 4.30) and bright blue blobs (*Z-score ≤ −4.30)*, respectively, indicate regions where bright pixels were significantly correlated positively with the judgment and regions where dark pixels were significantly correlated negatively with the judgment (Pixel test, two-tailed, search region = 4275 pixels, *p* < 0.05).

Next, for each type of judgment, we subtracted the CI associated with the group of young adults from the CI associated with the group of older adults, and divided by square root of 2 to transform into *Z*-scores. Pixel tests revealed no significant difference between the two groups. This is not too surprising because such a contrast is very conservative. Suppose, for example, that some pixels of the mouth attained statistical significance with a *Z*-score of 5 in a young CI and that it did not attain statistical significance with a *Z*-score of 0 in the corresponding old CI. The contrast CI would not attain significance [i.e., (5–0)/√2 = ~3.54, which is clearly below the statistical threshold of 4.30]. In fact, the young and old adults CIs were highly correlated: the proportion of shared variance (i.e., squared Pearson correlation) between the happiness CIs, within an ellipse of 63 × 95 pixels, or 3.35 × 5.05° of visual angle, centered on the faces, is 0.46 [95% confidence interval = (0.426, 0.488); all confidence intervals reported in this article were evaluated using a Bootstrap method; for details, see DiCiccio and Efron, 1996], that between the anger CIs is 0.36 [95% confidence interval = (0.327, 0.388)], and that between the trust CIs is 0.28 [95% confidence interval = (0.258, 0.306)]. These *R*^2^ values are all the more impressive that simulations put the maximum proportion of shared variance between the true CIs and the CIs derived in our experiment in the vicinity of 0.80 for old adults, and of 0.88 for younger adults. In turn, this imposes an upper limit on the proportion of shared variance between the measured smooth CIs in the young and older adults of about 0.70. In other words, the proportion of shared variance between the two trustworthiness CIs—0.28—should be compared to this maximum value of 0.7 rather than 1.

These similarities camouflage important differences between the two age groups. We measured the strength of relationship between the trustworthiness, happiness and anger CIs within the two subject groups. For the group of young adults, the trustworthiness CI is more like the happiness CI than the anger CI. Indeed, the shared variance between the trustworthiness and the happiness CIs is 0.35 [95% confidence interval = (0.320, 0.385)], again, within an ellipse of 63 × 95 pixels, whereas the shared variance between the trustworthiness and the anger CIs is 0.21 [95% confidence interval = (0.182, 0.235)]. However, these statistics are somewhat misleading because the happiness and the anger CIs shared an important proportion of their variances—0.37 [95% confidence interval = (0.339, 0.401)]. The proportion of shared variance between the trustworthiness and the happiness CIs, once the shared variance between the happiness and the anger CIs has been removed from the happiness CI [i.e., happiness CI - (a ^*^ anger CI + b), with a and b two scalars obtained by least-square linear regression] is then 0.16 [95% confidence interval = (0.141, 0.178)]; and the proportion of shared variance between the trustworthiness and the anger CIs, once the shared variance between the happiness and the anger CIs has been removed from the anger CI, is 0.014 [95% confidence interval = (0.0096, 0.0197)].

For the group of older adults, a slightly different pattern is observed. The shared variance between the trustworthiness and the happiness CIs is very similar to the shared variance between the trustworthiness and the anger CIs. Indeed, the proportion of shared variance between the trustworthiness and the happiness CIs is 0.30 [95% confidence interval = (0.277, 0.336)], whereas that shared variance between the trustworthiness CI and the anger CI is also 0.30 [95% confidence interval = (0.272, 0.328)]. But the happiness and the anger CIs shared as much as a proportion of 0.44 of their variances [95% confidence interval = (0.406, 0.473)]. The proportion of shared variance between the trustworthiness and the happiness CIs, once the shared variance between the happiness and the anger CIs has been removed from the happiness CI, is 0.06 [95% confidence interval = (0.054, 0.076)]; and the proportion of shared variance between the trustworthiness and the anger CIs, once the shared variance between the happiness and the anger CIs has been removed from the anger CI, is also 0.06 [95% confidence interval = (0.048, 0.069)].

## Discussion

The main purpose of this study was to investigate and compare implicit representations of faces expressing trustworthiness, happiness and anger in young and older adults using a reverse correlation method. To our knowledge, our study is the first one to use reverse correlation with older individuals in order to better understand processes underlying social judgments in this age group. Our study reveals that the internal representations of trust (28% of shared variance), anger (36% of shared variance), and happiness (46% of shared variance) are very similar for young and older adults. However, a more subtle analysis revealed that the relationship between the judgment of trust and the judgments of happiness and anger was different in young and in older adults. When we look at the results of the older group of participants, we find a large shared variance between happiness and anger CIs (42%), suggesting a continuum between the two representations, which is compatible with Todorov';s theory of the processes underlying trust judgments. Moreover, we find a large and equally shared variance (30%) between trust and anger, and trust and happiness, which, considered together, suggest that trust is judged on this continuum. This notion is reinforced by the findings that explained variances in trust drops by 80%, symmetrically (from 30 to 6%) once the shared variance (i.e., the continuum) between the happiness and anger CIs has been removed. In sum, in older adults, there seems to be an anger-happiness continuum, and this continuum seems to explain a large portion of the trust CI. Nonetheless, the shared variances between trust and the happiness and anger residuals suggest that there is more to the judgment of trust than simply the happiness-anger continuum.

In younger adults, the situation is somewhat different: again there is a large shared variance between happiness and anger CIs (37%), which suggests some sort of continuum between the two representations. However, the somewhat asymmetric shared variance between trust and happiness (35%) and trust and anger (21%), suggests that trust judgments made in the context of money investment are not only explained by this happiness-anger continuum, and that trust is more related to happiness than to anger. This is strengthened by the fact that 16% of shared variance remains (almost half) between trust and happiness once the happiness and anger shared variance (i.e., the continuum) has been removed from the happiness CI, whereas it';s only 1% for trust and anger only. In other words, for young adults, there is something specific about the representation of happiness that explains the trust representation in addition to the happiness-anger continuum, and very little, if anything, specific about anger that explains the trust representation.

The discrepancies between our results with young adults and the Todorov group results in many experiments exploring trust judgments in young adults should not be blown out of proportion. These discrepancies might originate from several methodological differences. One of the important factors to consider is that the stimuli we used were faces of women only. The connection between masculinity and anger, for example, could be stronger than the one between femininity and anger, which could explain why our group of young adults relied less on the anger judgment to make their trust judgment. This might explicate why the eyebrows are almost absent in our anger CIs while they have been shown to be preponderant in the anger CIs of Western Caucasian participants (Jack et al., 2011). Other differences in methodology could also explain differences in the results. Most importantly, we asked participants to judge trustworthiness in a specific context (i.e., “If you had a big amount of money to invest, who would you trust the most with your money?”) in order to eliminate context as a potential source of variability—especially across age groups—from our results.

Of course, social judgments are complex and multidimensional, and judgments of trust are no exception to this statement. Todorov';s theory inserts trustworthiness judgments in an emotional continuum, between anger and happiness. However, other researchers have positioned this judgment on different continuums. For instance, Fiske et al. (2007) have argued that social judgments can be classified in two main dimensions: warmth and competence. Judgments of warmth (i.e., perceived intent: friendliness, helpfulness, sincerity, morality, trustworthiness) would occur first in order to determine whether the person has good or bad intentions. Judgments of competence (i.e., perceived ability: intelligence, skill, creativity) would appear shortly after and would help determine whether the person has the ability to act these intentions. These two dimensions would be linked and positively correlated. Even though our goal was to diminish ambiguity concerning the type of judgment asked, judging trust in a context of financial investment could have led to associating trust in a stronger way to a competence type of judgment than to a warmth type of judgment. However, the dimensions of warmth and competence, as described by Fiske et al. (2007), are positively correlated, which suggests that trustworthiness judgments could be embedded in both categories.

One problem in studying trustworthiness judgments is that it is very difficult to establish a baseline allowing a “true” value of trustworthiness. For example, when studying emotional judgments, it is possible to take photographs of people feeling real emotions, thereby allowing us to know that a given photograph of someone expressing a feeling of joy really is a true and valid representation of the emotion. In trust judgments, it seems impossible to find photographs of people that we know for sure can be trusted, and then to classify objectively how trustworthy these people are, based on their life experiences. This difficulty thus makes it hard to establish a measure of performance in trustworthiness judgments and to rely on this measure in order to study variability in the way different people judge how trustworthy faces are. Since judgments of trustworthiness are of particular importance in our everyday interactions, it seems necessary to generate new ways to explore this question more efficiently, particularly in more vulnerable populations such as older adults.

### Conflict of interest statement

The authors declare that the research was conducted in the absence of any commercial or financial relationships that could be construed as a potential conflict of interest.
